# Widespread evolutionary crosstalk among protein domains in the context of multi-domain proteins

**DOI:** 10.1371/journal.pone.0203085

**Published:** 2018-08-31

**Authors:** David Jakubec, Miroslav Kratochvíl, Jiří Vymĕtal, Jiří Vondrášek

**Affiliations:** 1 Department of Bioinformatics, Institute of Organic Chemistry and Biochemistry of the Czech Academy of Sciences, 166 10 Prague 6, Czech Republic; 2 Department of Physical and Macromolecular Chemistry, Faculty of Science, Charles University, 128 43 Prague 2, Czech Republic; 3 Department of Software Engineering, Faculty of Mathematics and Physics, Charles University, 118 00 Prague 1, Czech Republic; Indian Institute of Science, INDIA

## Abstract

Domains are distinct units within proteins that typically can fold independently into recognizable three-dimensional structures to facilitate their functions. The structural and functional independence of protein domains is reflected by their apparent modularity in the context of multi-domain proteins. In this work, we examined the coupling of evolution of domain sequences co-occurring within multi-domain proteins to see if it proceeds independently, or in a coordinated manner. We used continuous information theory measures to assess the extent of correlated mutations among domains in multi-domain proteins from organisms across the tree of life. In all multi-domain architectures we examined, domains co-occurring within protein sequences had to some degree undergone concerted evolution. This finding challenges the notion of complete modularity and independence of protein domains, providing new perspective on the evolution of protein sequence and function.

## Introduction

**Fig 1:**
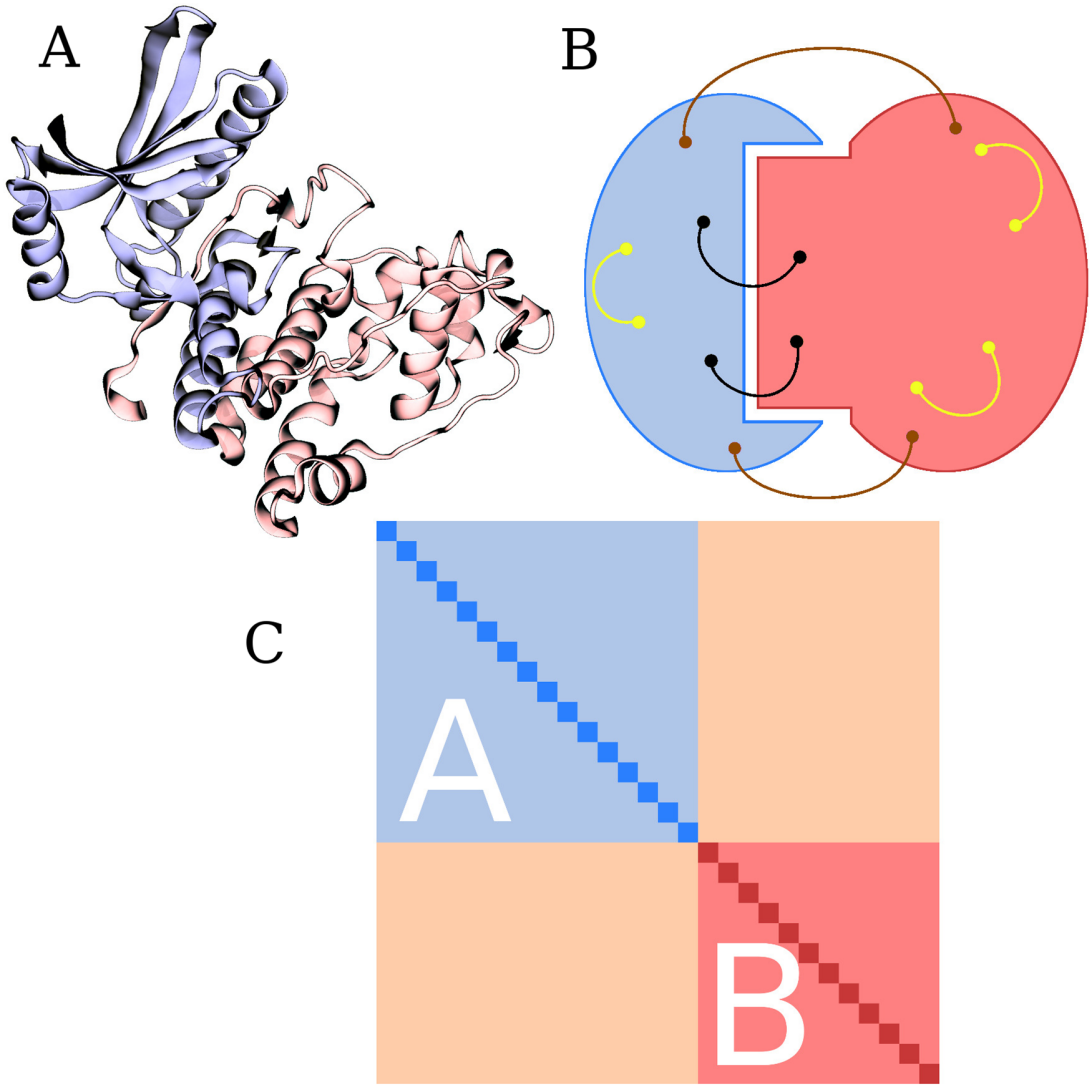
Representations of a two-domain architecture. A: Example structure of a two-domain protein. Chain A in Protein Data Bank [18] entry 1WAK contains two PF00069 (protein kinase) domains, shown here in blue and red. B: Correlated mutations in multi-domain proteins. Blue and red areas schematically depict individual domains involved in an interaction. Dots connected by solid lines represent pairs of coevolving positions. Coevolving positions within individual domains are shown in yellow. Coevolving positions localized in different domains are shown in black and brown. These can involve both positions forming physical contacts at the inter-domain interface (black) as well as positions separated by large distances (brown). C: General structure of a mutual information matrix for a two-domain architecture *AB*. White A and B labels denote individual domains. Diagonal dark blue and dark red elements describe the entropy of individual positions within domain *A* or *B*, respectively. Off-diagonal light blue and light red elements describe the mutual information between pairs of positions within domain *A* or *B*, respectively. Off-diagonal pink elements describe the mutual information between pairs of positions belonging to different domains.

Domains are basic functional and structural elements of proteins. In addition to sequence mutations, protein evolution is driven by combining existing domains into novel arrangements. The modular nature of domains arises from their ability to adopt well-defined three-dimensional (3D) structures (Fig 1A) that often facilitate their functions independently of their sequential surroundings. [1–3] Most eukaryotic proteins contain multiple domains [4, 5], and interactions among these domains can mediate allosteric regulation [6] or give rise to novel domain functions different from those found in isolation or other domain arrangements.

Structural changes driven by mutations in the primary sequence are one mechanism underlying the acquisition of novel domain functions. [4, 7, 8] Structural and functional constraints often require that evolution be coordinated between groups of amino acid residues in proteins (Fig 1B). [9–11] Covariation in amino acid composition between positions in multiple sequence alignments (MSAs) can be indicative of physical interactions between the residues and has been used to aid prediction of protein 3D structures and conformational diversity. [12–17] Massive utilization of coevolutionary information has been made possible recently by the availability of high-quality MSAs containing data from high-throughput sequencing experiments. [14, 16, 17, 19, 20] Coevolutionary signals have been described both within and among domains that coexist within protein chains. [12, 21] In addition, coevolution has been also observed between proteins and their protein or nucleic acid interacting partners. [22–24]

In this work, we present an information-theoretic analysis of coevolutionary signals among protein domains in multi-domain arrangements. Based on the functional implications these signals carry, we test the notion of evolutionary and functional independence of domains and examine their adaptability to their primary sequence context. In contrast to the aforementioned studies, we examine coevolution as a global property of a domain pair, and introduce an appropriate continuous measure to quantify its effect. Using this measure, we show that coevolution among protein domains is a much more widespread phenomenon than previously anticipated.

## Materials and methods

### Data set construction

We defined protein domains as sequence families recognized in release 31.0 of the Pfam database. [19] Pfam 31.0 provides a collection of 16,712 profile hidden Markov models (HMMs), each representing one protein sequence family, as well as MSAs containing alignments of sequences in UniProtKB [20] and other databases to these profile HMMs. Alignments of sequences included in release 2016_10 of the UniProt reference proteomes (URPs) to the Pfam 31.0 profile HMMs were obtained from the Pfam FTP repository. This URPs release contains a total of 26,742,727 protein sequences comprising the proteomes of 6,266 completely sequenced organisms. At least one match to a Pfam 31.0 profile HMM was recognized in 19,419,549 of these sequences. A total of 16,479 Pfam 31.0 profile HMMs matched to at least one sequence from the URPs.

Domain architecture was established for each URPs sequence. We defined the domain architecture of a protein as a vector of Pfam 31.0 sequence families identified within the protein sequence ordered according to their proximity to the N-terminus. A total of 278,458 distinct domain architectures were recognized. In order to reduce small-sample effects [25, 26], only architectures realized in at least 500 URPs sequences were considered; a total of 2,063 such architectures contained two or more domains and were thus selected for this study. A total of 4,240,857 URPs sequences were recognized as having one of these highly populated multi-domain architectures (HPAs). A total of 2,599 distinct Pfam 31.0 sequence families were identified within these sequences.

For each HPA, we compiled a list of all URPs sequences in which it was realized. Sequences of domains found in these proteins aligned to the respective Pfam 31.0 profile HMMs were retrieved from the Pfam family MSAs. Proteins that contained only standard amino acid, insert, and delete state symbols in the alignments of sequences of each of their domains to the respective Pfam 31.0 profile HMMs were identified for all HPAs. The lists of UniProt identifiers of these proteins for individual architectures are available in S1 File; the distribution of the numbers of sequences with these architectures is shown in S1 Fig.

Residue symbols found at positions corresponding to match or delete states (consensus columns) in the alignments of the respective domain sequences to the profile HMMs were extracted from each domain sequence within these proteins. This action corresponds to localizing all but the insert state positions in the respective MSAs, as residues assigned to insert states are, by definition, unaligned, and therefore irrelevant to this study. [27] Sequences of domains composed of the residues found in the consensus columns were then concatenated for each protein, creating a string composed of residues characteristic of each domain identified within the URPs sequence. For example, if protein *i* contained two domains *A* and *B* with respective sequences *A_i_* and *B_i_*, the concatenated sequence *A_i_*‖*B_i_* was created. By generating this string for each protein, we created a multi-domain MSA for each HPA.

### Normalized mutual information measure

Individual columns in a MSA can be viewed as random variables, with residue symbols found in the columns acting as the values of their respective observations. The Shannon entropy H(*X*) of a random variable *X* taking on values from a finite alphabet $K=\{x_{1},x_{2},\ldots ,x_{K}\}$ can be estimated as

$$\begin{array}{c} H\left(X\right)=-\sum_{i=1}^{K}f\left(x_{i}\right)\log_{2}f\left(x_{i}\right), \end{array}$$ (1)

where *f*(*x*_*i*_) is the relative frequency of observing *x*_*i*_ and 0 log_2_ 0 is defined as zero. The mutual information MI(*X*, *Y*) of a pair of random variables *X*, *Y* can be estimated as

$$\begin{array}{c} \text{MI}\left(X,Y\right)=\sum_{i=1}^{K}\sum_{j=1}^{L}f\left(x_{i},y_{j}\right)\log_{2}\frac{f(x_{i},y_{j})}{f\left(x_{i}\right)f\left(y_{j}\right)}, \end{array}$$ (2)

where *f*(*x*_*i*_, *y*_*j*_) is the joint frequency of observing *x*_*i*_ and *y*_*j*_ simultaneously. Since 2 is chosen as the base of the logarithms in Eqs 1 and 2, values of entropy and mutual information are in bits. [28] Throughout this work, *K* and *L* are equal to 21, as all sequences in the MSAs contain only symbols for the standard amino acids and the delete state.

**Fig 2:**
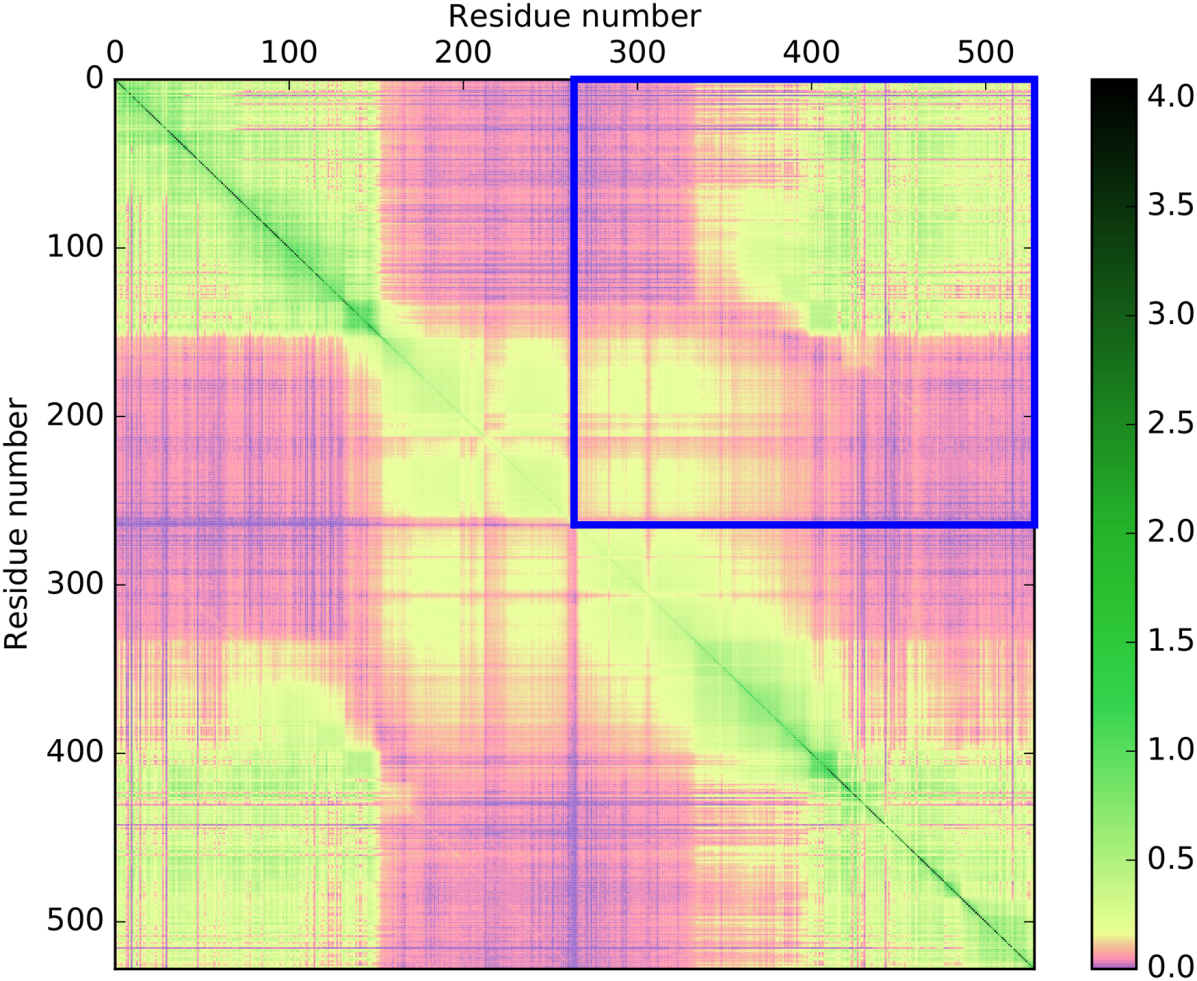
Mutual information matrix for the native PF00069–PF00069 (protein kinase–protein kinase) two-domain architecture. Each protein kinase domain contains 264 residues. A total of 8,753 URPs sequences have this architecture. The respective MSA consists of sequences of the two domains found within each of these URPs sequences. Note the non-linear color scale and the positive values of mutual information between positions corresponding to different domains (highlighted with the blue square). Values of entropy and mutual information are in bits.

A mutual information matrix (MIM) showing the values of mutual information between each pair of columns within a MSA was calculated for multi-domain MSAs corresponding to the 2,063 selected multi-domain HPAs. The general structure of a MIM for a two-domain architecture is shown in Fig 1C, and an example of a MIM for an architecture consisting of two protein kinase (Pfam entry PF00069) domains is shown in Fig 2. In addition to correlated mutations within individual domains, these representations reveal positive values of mutual information between positions corresponding to different domains.

We calculated the average entropy ${\bar{H}}_{D}$ of positions corresponding to domain *D* for each domain within each HPA as

$$\begin{array}{c} {\bar{H}}_{D}=\frac{1}{n}\sum_{i=1}^{n}H\left(X_{i,D}\right), \end{array}$$ (3)

where *n* is the number of positions *X_i,D_* corresponding to domain *D*. This corresponds to calculating the average values of the dark blue or dark red elements for the MIM shown in Fig 1C.

There are a total of (N2) unique domain pairs for an architecture consisting of *N* domains. For all such pairs of domains *D*, *E* within each architecture (regardless of whether they are sequential neighbors or not), the average value of mutual information $\bar{MI}\equiv {\bar{MI}}_{D,E}$ between positions corresponding to the two domains was calculated as

$$\begin{array}{c} {\bar{\text{MI}}}_{D,E}=\frac{1}{mn}\sum_{i=1}^{m}\sum_{j=1}^{n}\text{MI}\left(X_{i,D},Y_{j,E}\right), \end{array}$$ (4)

where *m*, *n* are the numbers of positions *X_i,D_*, *Y_j,E_* corresponding to domains *D* and *E*, respectively. This corresponds to calculating the average value of matrix elements in pink rectangles in the general MIM shown in Fig 1C.

We calculate the normalized average inter-domain mutual information $n\bar{MI}\equiv n{\bar{MI}}_{D,E}$ as a ratio of the average inter-domain mutual information ${\bar{MI}}_{D,E}$ and the arithmetic average of average entropies of positions corresponding to domains forming the respective pair, *i.e*.,

$$\begin{array}{c} n{\bar{\text{MI}}}_{D,E}=\frac{2}{{\bar{H}}_{D}+{\bar{H}}_{E}}{\bar{\text{MI}}}_{D,E}. \end{array}$$ (5)

The value of $n\bar{\text{MI}}$ obtained in this way is equivalent to the statistical measure known as symmetric uncertainty. [29] It represents, on the scale from 0 to 1, the extent of evolutionary coupling between domains *D* and *E*, independent of the internal sequence variability of each domain. There were a total of 5,205 domain pairs for which the value of $n\bar{\text{MI}}$ was calculated according to Eq 5.

### Information-theoretic analysis

To compare the extent of evolutionary crosstalk among protein domains in the selected multi-domain architectures with the crosstalk in sequences that share no evolutionary history, we have designed the following test. First, we split strings consisting of concatenated domain sequences at the domain boundaries. If domain architecture *AB* was found in *N* protein sequences and the corresponding multi-domain MSA contained sequences *A*_1_‖*B*_1_, *A*_2_‖*B*_2_, …, *A_N_*‖*B_N_*, then two sets of sequences, {*A*_1_, *A*_2_, …, *A_N_*} and {*B*_1_, *B*_2_, …, *B_N_*}, were obtained after the original sequences had been split. After this splitting, sequences of individual domains within these sets were then randomly shuffled and rejoined so that the original architecture was reestablished. For example, if protein *i* with domain architecture *AB* originally contained a concatenated domain sequence *A_i_*‖*B_i_*, the shuffling process resulted in the sequence *A_i_*‖*B_j_*, with *j* being a different URPs sequence with an *AB* architecture. This way, any native evolutionary coupling among protein domains was disrupted.

**Fig 3:**
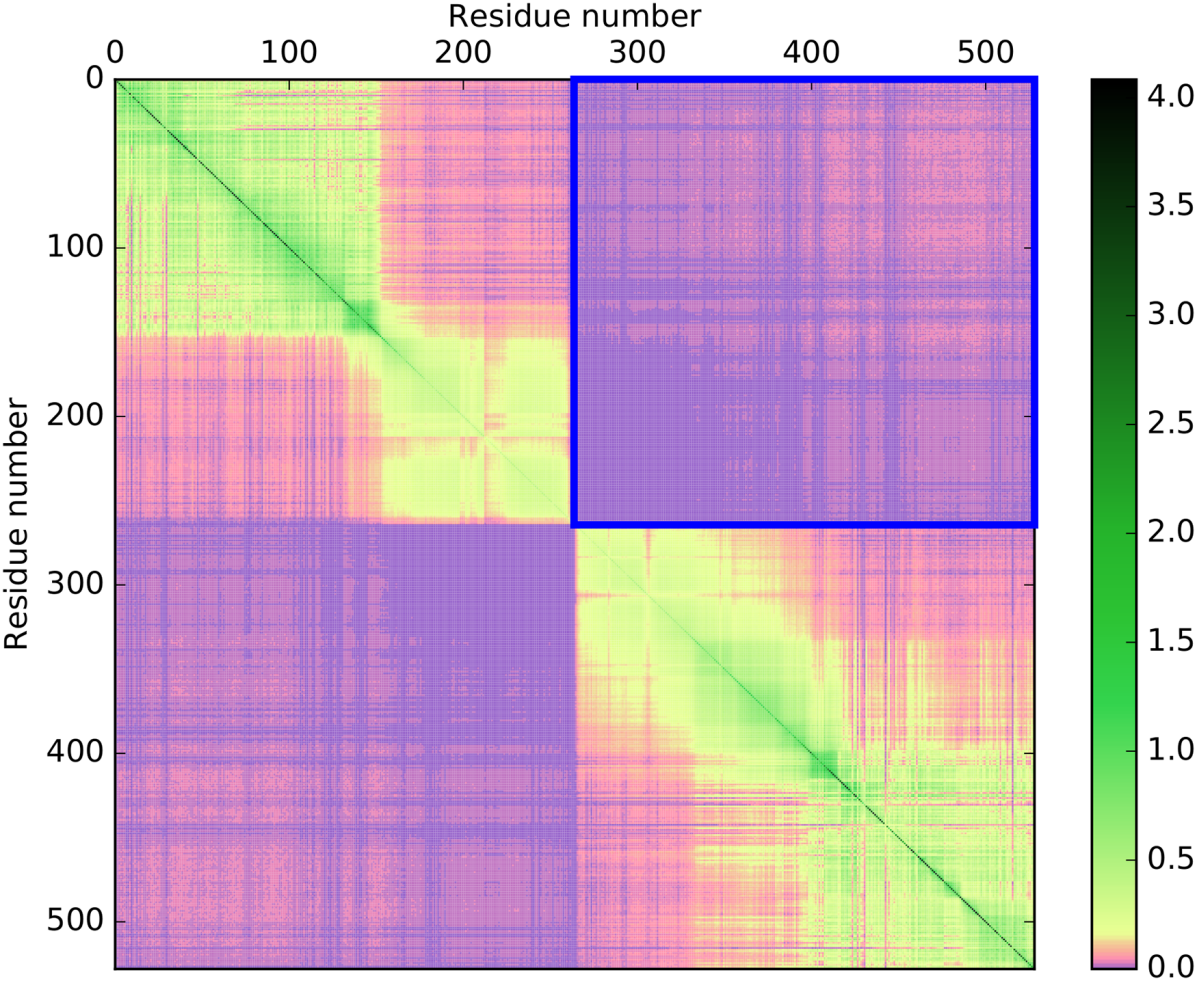
Mutual information matrix for the perturbed PF00069–PF00069 (protein kinase–protein kinase) domain architecture. Sequences of individual domains were randomly shuffled and rejoined. Note how the mutual information between positions corresponding to different domains (blue square) has vanished. Values of entropy and mutual information are in bits. The color scale is the same as in Fig 2.

For each HPA, we calculated a second MIM from the perturbed MSA of disrupted sequences in which fragments corresponding to individual domains almost certainly originated from different proteins. For each pair of domains in each architecture, the value of $n\bar{\text{MI}}$ was calculated based on the respective perturbed MSA as described above for the case of native sequences. An example of such a MIM for the architecture consisting of two protein kinase (PF00069) domains is shown in Fig 3. Here, one can clearly see that the inter-domain mutual information decreased remarkably in comparison with Fig 2.

To further examine the influence of background information noise arising among uncorrelated sequences, we studied the effects of random sequence composition fluctuations on the resulting values of $n\bar{\text{MI}}$. A total of 100,000 amino acid sequences, each containing 100 randomly selected residues, were generated. The probabilities of choosing individual amino acids were the same at each position within the sequences and were obtained from the average primary sequence composition of proteins in release 2017_08 of the UniProtKB/Swiss-Prot database. [20] For each of the 2,599 unique Pfam 31.0 sequence families identified within the HPAs, 500 sequences were randomly chosen from the corresponding MSA of domain sequences. Each of these sequences was then concatenated with a randomly chosen sequence from the set of 100,000 sequences with random composition. In this way, a sort of a two-domain MSA was generated, in which very high-entropy positions correspond to one of the domains. The value of $n\bar{\text{MI}}$ was then calculated for each of these pseudoarchitectures.

## Results

**Fig 4:**
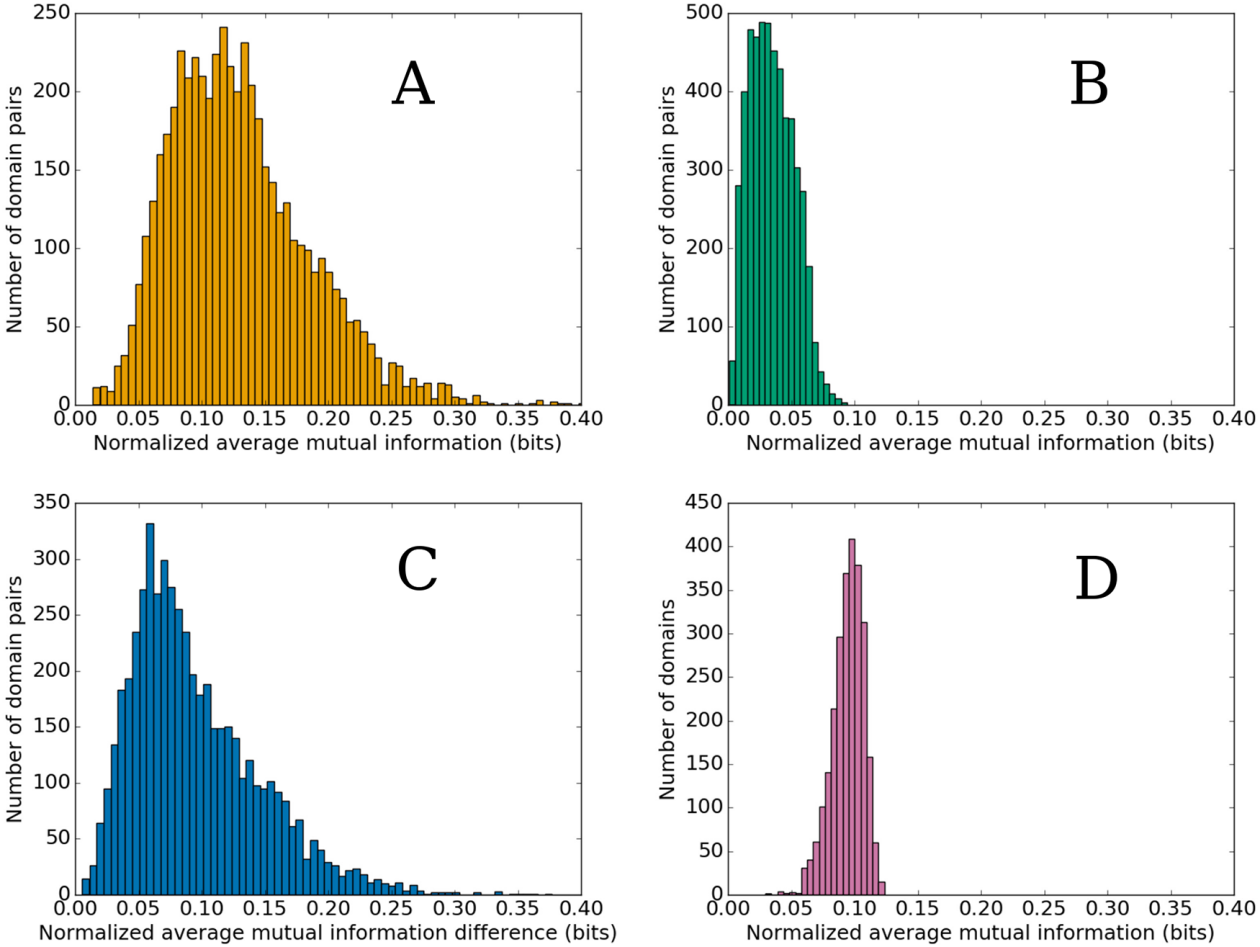
Distributions of the values of $n\bar{\text{MI}}$ for various domain pairings. A: Distribution of the values (*N* = 5, 205) of $n\bar{\text{MI}}$ for domain pairs in native (non-disrupted) multi-domain protein sequences. B: Distribution of the values (*N* = 5, 205) of $n\bar{\text{MI}}$ for domain pairs after intra-architectural domain sequence shuffling. C: Distribution of differences (*N* = 5, 205) between the values of $n\bar{\text{MI}}$ for individual domain pairs before and after intra-architectural domain sequence shuffling. D: Distribution of the values (*N* = 2, 599) of $n\bar{\text{MI}}$ for domain–random sequence pairs.

There were a total of 5,205 domain pairs for which the values of $n\bar{\text{MI}}$ were calculated before and after intra-architectural domain sequence shuffling. The distributions of resulting $n\bar{\text{MI}}$ values are shown in Fig 4A and 4B, respectively; the raw obtained values are available in S1 File. The difference between these values was calculated for each domain pair. The distribution of these differences is shown in Fig 4C. For illustration, the value of this difference is ≈ 0.068 bits for the exemplary PF00069–PF00069 two-domain architecture.

It is clear from Fig 4C that all differences between the values of $n\bar{\text{MI}}$ before and after intra-architectural domain sequence shuffling are greater than zero, *i.e*., the $n\bar{\text{MI}}$ is always greater before the domain sequence shuffling. As a hypothesis, this is confirmed by performing the Wilcoxon signed-rank test [30] on the distribution of these differences, which yields both the value of the test statistic and the *p*-value of 0.0. The difference between the distributions shown in Fig 4A and 4B can also be confirmed by performing the two-sided two-sample Kolmogorov–Smirnov (KS) test [31], which yields the value of the KS statistic of ≈0.869 and the corresponding *p*-value effectively zero.

It should be noted that both the distribution of the values of $n\bar{\text{MI}}$ in native sequences (Fig 4A) and the distribution of the $n\bar{\text{MI}}$ differences (Fig 4C) have means of around 0.1 bits, which can be considered significant, given that maximum entropy of individual positions in the MSAs is on the order of 10^0^ bits.

Fig 4D shows the distribution of the values of $n\bar{\text{MI}}$ for sequences consisting of natural domains and random sequences. Here, one can see that the distribution differs significantly from that observed for native multi-domain sequences (Fig 4A) and appears to be more similar to the distribution obtained after domain sequence shuffling (Fig 4B). However, a deeper statistical inspection performed using the two-sided two-sample KS test and the two-sided Mann–Whitney *U* test [32] shows that both non-random distributions are significantly different (all *p*-values are effectively zero). This result shows that, even though each individual position in the artificial random sequences has nearly maximum possible entropy, and thus has a large potential to generate considerable values of mutual information with positions in genuine protein sequences due to statistical noise, the observed values of $n\bar{\text{MI}}$ differ significantly from those observed in natural multi-domain architectures. Therefore, it seems unlikely that the evolutionary coupling observed among domains in genuine multi-domain proteins would be a result of random fluctuations in amino acid residue frequencies.

**Fig 5:**
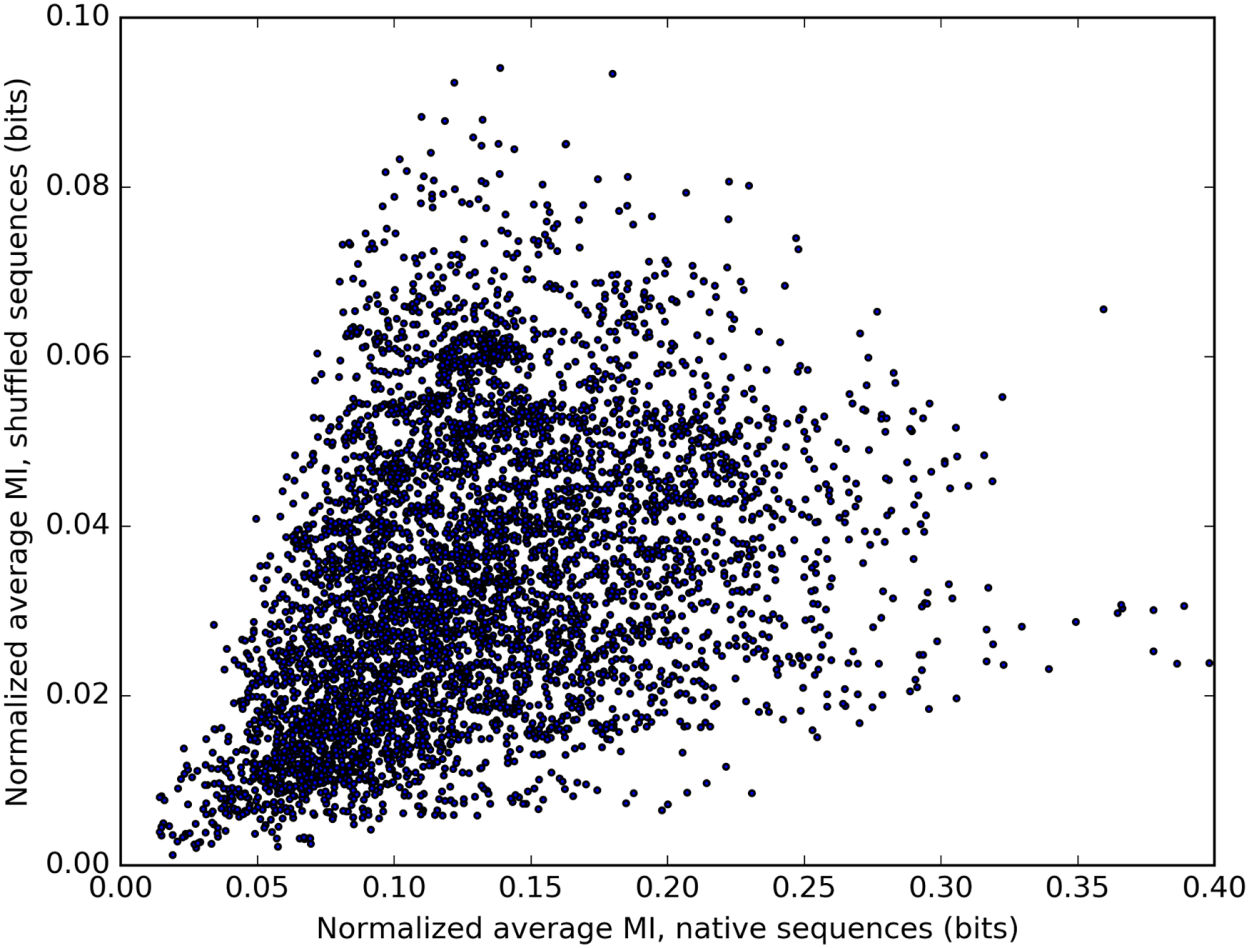
Correlation between the values of $n\bar{\text{MI}}$ before and after intra-architectural domain sequence shuffling. Number of domain pairs (data points) *N* = 5, 205. The value of the Pearson correlation coefficient *r* ≈ 0.345; the value of the Spearman’s rank correlation coefficient *ρ* ≈ 0.432.

**Fig 6:**
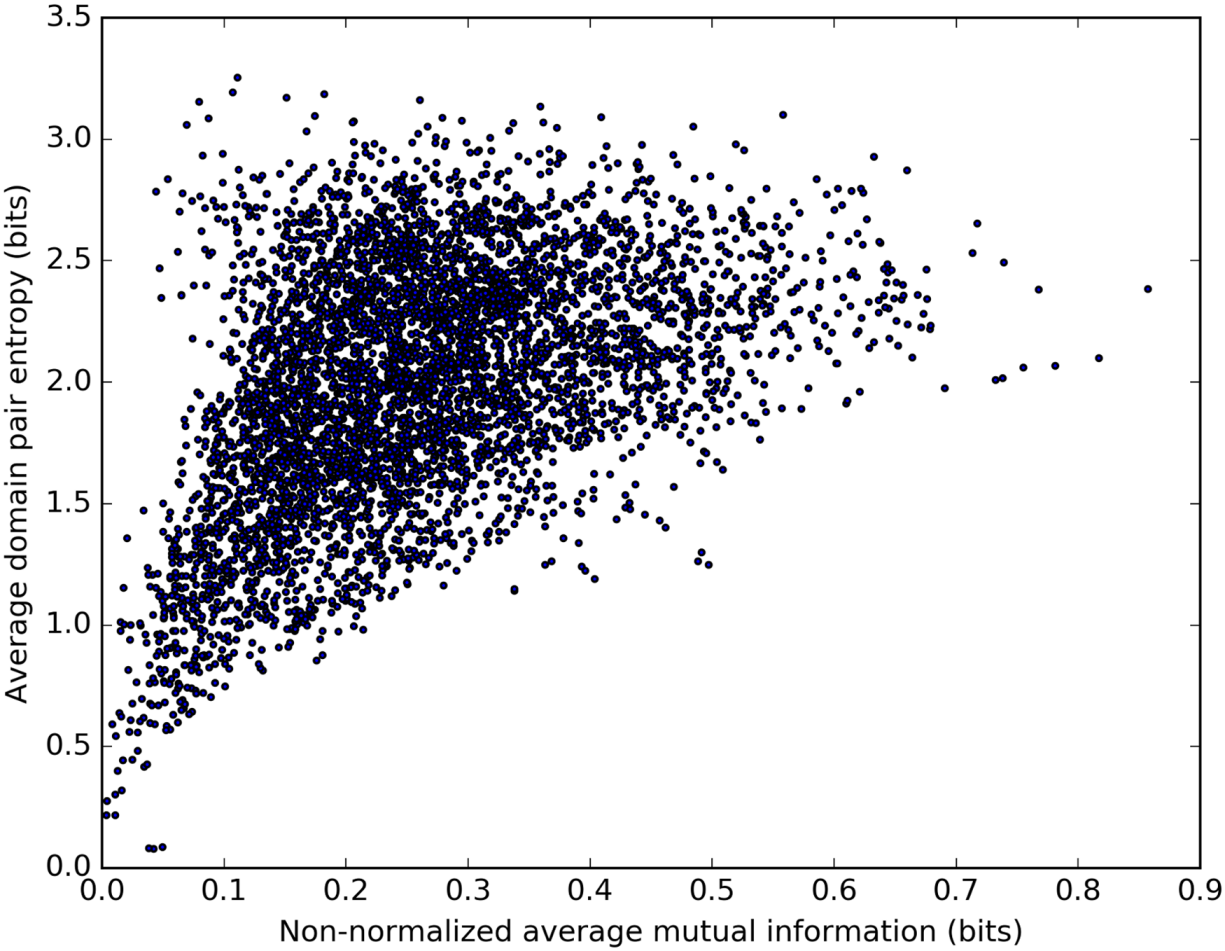
Correlation between the non-normalized values of $\bar{\text{MI}}$ and average domain pair entropies. Number of domain pairs (data points) *N* = 5, 205. The value of the Pearson correlation coefficient *r* ≈ 0.464; the value of the Spearman’s rank correlation coefficient *ρ* ≈ 0.460.

In addition, the apparent similarity of the distributions shown in Fig 4B and 4D implies that the domain sequence shuffling procedure has reduced the inter-domain sequence covariation score almost to the level expected to result from statistical noise. However, it should be noted that there is a weak–intermediate positive linear correlation between the values of $n\bar{\text{MI}}$ before and after domain sequence shuffling for individual domain pairs (Fig 5). Therefore, it seems that not all contained information could be eliminated using this approach. This could be related to a similar positive linear correlation observed between the non-normalized values of $\bar{\text{MI}}$ (Eq 4) and the respective average domain pair entropies $\frac{{\bar{H}}_{D}+{\bar{H}}_{E}}{2}$ (Fig 6). We provide an explanation for the observation of these correlations in Discussion.

It is worth noting that the values of $n\bar{\text{MI}}$ and the respective average domain pair entropies are virtually uncorrelated (Pearson correlation coefficient *r* ≈ −0.001; Spearman’s rank correlation coefficient *ρ* ≈ −0.005).

## Discussion

While most studies of coevolution to date (for example, [11–17, 21–24] and many others) have treated it as a discrete property, in this paper we study the degree of coevolution as a continuous property. Unlike the mentioned studies, our aim has not been the identification of precise “coevolving” pairs of residues in close spatial proximity, but rather the mapping of the overall tendency of residues in a pair of domains to respond to mutations in their partner. The value of $n\bar{\text{MI}}$ introduced here is an intrinsically global measure which quantifies this effect for a pair of domains without specifying which precise residue pairs contribute to this measure significantly. This makes it serve a different and unique role compared to recent coevolutionary analyses for 3D contact prediction, such as DCA [12] and PSICOV. [33]

In this analysis, we deliberately ignored the issue of transitivity, where two domains may appear to be coevolving if they share a common partner which is coevolving with both of them. It may not be possible to reliably quantify and filter out such effects from purely numerical data when coevolution is treated as a continuous property and some signal is observed for all pairs of domains (a possible linear optimization-based solution is highly numerically unstable). In addition, a pair of domains could, in principle, be coevolving with some other conserved non-domain region, such as a linker, and these effects could not be filtered out even if an appropriate method existed. It is worth noting that most (1,390 out of 2,063) architectures studied in this work consist of only two protein domains, which rules out the possibility of domain-related transitivity effects.

We explain the correlation between the values of $n\bar{\text{MI}}$ before and after domain sequence shuffling, and the related correlation between the non-normalized values of $\bar{\text{MI}}$ and the respective average domain pair entropies as follows. When two variables (positions in a MSA) each have a large entropy, there is a greater chance that mutual information will appear between the variables due to random noise, as mutual information can only increase with increasing entropy. Therefore, if one calculates the value of $\bar{\text{MI}}$ for a pair of domains with large average entropies, one can expect the result to be greater as a consequence of an increased statistical noise (Fig 6). As the intra-architectural domain sequence shuffling has no effect on entropies of individual positions, the increased chance to generate mutual information from random noise remains unchanged after domain sequences are shuffled. This mutual information can compensate some of the loss introduced by the domain sequence shuffling. Therefore, domain pairs with large average entropies can produce larger values of $\bar{\text{MI}}$ both in native and in shuffled multi-domain sequences, leading to the observed correlation (Fig 5).

In addition to the random noise factor described above, there is another contribution to the correlation observed in Fig 6, caused by the natural bounds on the value of mutual information between two variables. This value can never exceed the intrinsic entropy of either variable. Therefore, two positions with small entropies can never produce a large value of mutual information, whereas positions with large entropies may yield both small and large mutual information. This asymmetry can contribute to the observed correlation.

The value of $n\bar{\text{MI}}$ calculated for a pair of protein domains after intra-architectural domain sequence shuffling serves as a proxy for the value expected if domains from different proteins were paired randomly and thus shared no evolutionary history with each other. We found that the corresponding value of $n\bar{\text{MI}}$ calculated from the alignment of native (non-disrupted) multi-domain sequences is always greater (Fig 4C). This result implies that, in the context of multi-domain proteins, a portion of domain sequence variation can always be attributed to coordinated evolution among different domains.

Coordinated evolution can be intuitively understood by the need to preserve essential protein function in cases in which multiple domains form a ligand-binding or catalytic site (Fig 1B). [12, 21] We propose the following possible explanation for observation of this phenomenon even among domains lacking such apparent functional constraints: Co-localizing multiple domains into the same polypeptide chain may influence the folding pathway or open new paths to optimize protein function *via* inter-domain interactions. These interactions may enable direct or allosteric modulation of the function of the complete protein or its individual domains. If a vital function of a multi-domain protein depends on the cooperative action of its domains, evolution may opt to distribute mutations needed to preserve this function across the domains in a coordinated fashion. Residues preferentially mutated in this way may constitute nodes of energetic connectivity in the protein structure analogous to those observed at the single domain level. [34] It should be noted that $n\bar{\text{MI}}$, as defined, is a measure representing overall evolutionary coupling of two domains, and does not provide detailed insight into which specific amino acid residue pairs contribute significantly to this coupling.

In either case, additional domains act as buffers or reservoirs of evolutionary capacity that can be utilized to either mitigate the impact of mutations required to maintain proper protein function or, alternatively, to optimize the respective functions of individual domains. The precise mechanism through which this functional modulation is realized and its full impact on protein evolution remain to be established.

## Conclusion

We showed that, in the context of multi-domain proteins, evolution of domain sequences proceeds in a coordinated fashion. We proved this by comparing a mutual information-based measure between native multi-domain sequences and artificial sequence constructs which share no common evolutionary history and further showed that the observed evolutionary coupling is distinct from statistical noise.

## Supporting information

S1 FilePrimary data archive for the studied architectures.This archive contains a file presenting the list of UniProt identifiers of the URPs sequences included in the multi-domain MSAs for each of the 2,063 studied multi-domain architectures, and files containing the values of $\bar{\text{MI}}$ and $n\bar{\text{MI}}$ for individual domain pairs before and after domain sequence shuffling.(GZ)

S1 Fig Distribution of the numbers of sequences in the MSAs. Total number of architectures (MSAs) *N* = 2, 063.
(TIF)
