# Supplementary figures and images for: Widespread evolutionary crosstalk among protein domains in the context of multi-domain proteins
Source: PLoS One. 2018 Aug 31;13(8):e0203085. doi: 10.1371/journal.pone.0203085 (PMC6118372; doi:10.1371/journal.pone.0203085)

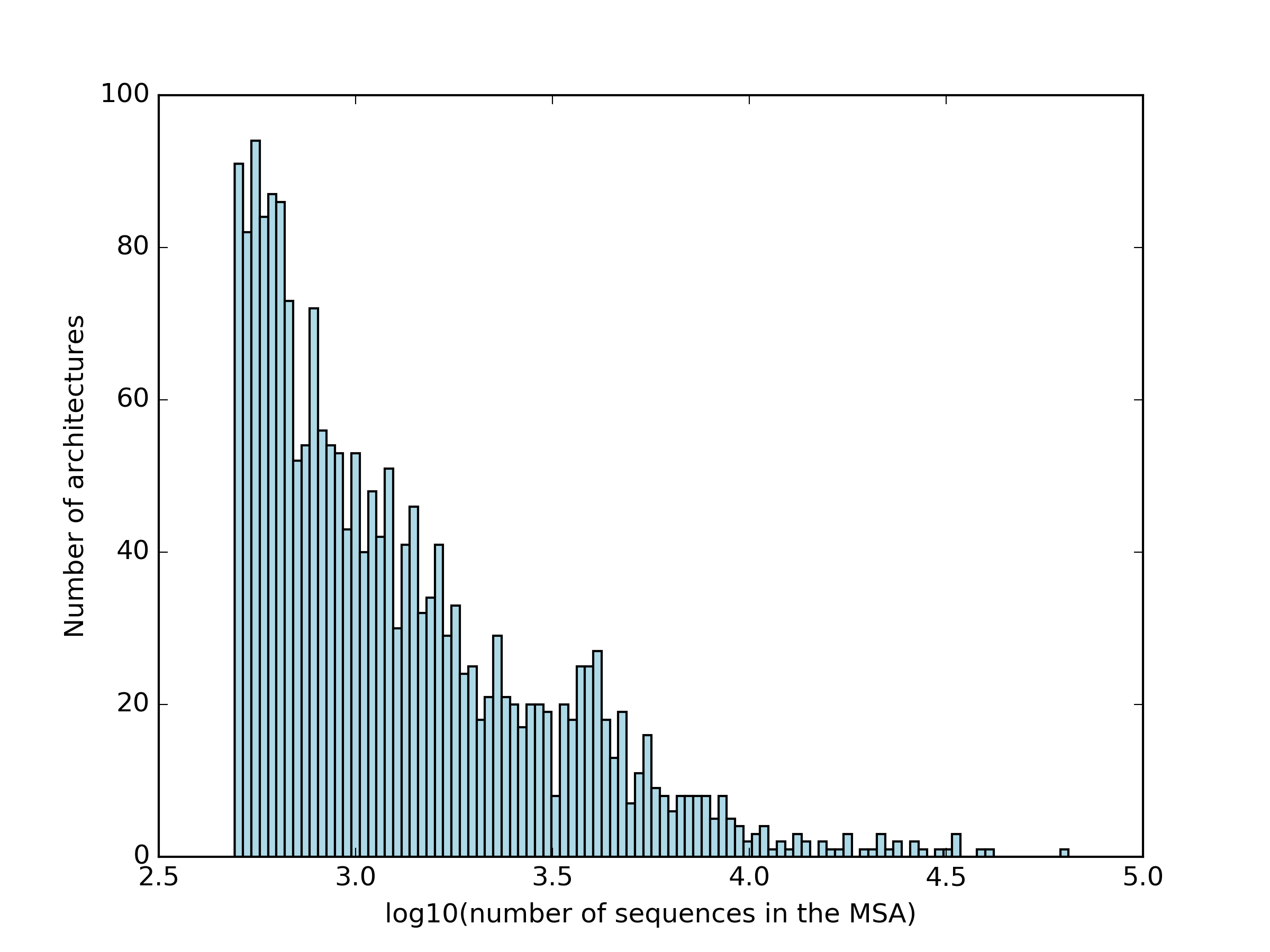

Supplement: S1 Fig — Total number of architectures (MSAs) N = 2, 063. (TIF) [file pone.0203085.s002.tif]
